# Calenduloside E alleviates cerebral ischemia/reperfusion injury by preserving mitochondrial function

**DOI:** 10.1007/s10735-022-10087-5

**Published:** 2022-07-12

**Authors:** Jianxiong Li, Yujie Bu, Bin Li, Hailin Zhang, Jia Guo, Jianping Hu, Yanfang Zhang

**Affiliations:** 1 Department of Neurology, Lanzhou University Second Hospital, 730030 Lanzhou, Gansu Province China; 2 Neurosurgery, Lanzhou University Second Hospital, 730030 Lanzhou, Gansu Province China

**Keywords:** Calenduloside E, Cerebral ischemia/reperfusion, Mitochondrial fission, Drp1, ROS

## Abstract

Calenduloside E (CE) isolated from *Aralia elata* (Miq.) Seem. is a natural triterpenoid saponin that can reportedly ameliorate myocardial ischemia/reperfusion injury. However, its potential roles and mechanism in cerebral ischemia/reperfusion injury are barely understood. In this study, we established an oxygen-glucose deprivation/reoxygenation (OGD/R) model in HT22 cells. We found that CE significantly attenuated the OGD/R-induced inhibition of cell viability and apoptotic cell death in HT22 cells. Moreover, CE treatment significantly ameliorated OGD/R-induced mitochondrial fission by inhibiting mitochondrial dynamin-related protein 1 (Drp1) recruitment and increasing Drp1 phosphorylation at Ser637. CE treatment significantly ameliorated OGD/R-induced mitochondrial dysfunction by increasing the mitochondrial membrane potential and reducing the mitochondrial ROS and cellular calcium accumulation. Moreover, CE treatment significantly inhibited the OGD/R-induced release of mitochondrial Cytochrome C and increase in Bax, Cleaved-caspase3 and Cleaved-caspase9 protein levels, whereas CE treatment significantly reversed the OGD/R-induced decrease in Bcl-2 and full length of caspase3 and caspase9 protein levels. In vivo, we found that CE treatment significantly ameliorated ischemic/hypoxic-induced brain infarct volume, neurological deficits, and neuronal apoptosis in mice after middle cerebral artery occlusion and reperfusion. CE treatment also significantly ameliorated the mitochondrial transmembrane potential, decreased Cytochrome C release, and reversed the increase in Bax, Cleaved-caspase3 and Cleaved-caspase9 protein levels and the decrease in Bcl-2 and full length of caspase3 and caspase9 protein levels induced by cerebral ischemia/reperfusion (I/R). All these results indicated that CE treatment exerted a neuroprotective effect by ameliorating mitochondrial dysfunction during cerebral I/R injury.

## Introduction

Stroke, one of the leading causes of death worldwide, is caused by the sudden blockage of blood flow or the rupture of blood vessels and can be divided into ischemic stroke and hemorrhagic stroke (Herpich and Rincon 2020; Shao et al. 2020; Stinear et al. 2020). Ischemic stroke accounts for approximately 70–85% of all strokes worldwide (Gąsecki et al. 2020). After cerebral ischemia, intravenous thrombolysis has been the most effective treatment for patients to date, but only a minority of patients can receive the treatment owing to its strict and narrow therapeutic window (less than 4.5 h) and the risk of intracranial bleeding (Liu et al. 2018b). Reperfusion can also restore blood supply, but this method also causes oxidative stress and inflammation that lead to accelerated neuronal damage (Tobin et al. 2014). Therefore, investigating the mechanisms underlying cerebral ischemia injury to reduce the associated adverse effects is essential. After ischemic stroke, neurons in the ischemic core die rapidly by necrosis, and the damage is irreversible. However, neurons in the transition or penumbra zone primarily undergo delayed apoptosis, and the damage is not irreversible (Zhou et al. 2018). Interestingly, the neuronal damage in the transition is slower, which can provide a therapeutic window to prevent brain-injury progression. Furthermore, the effects of neuronal apoptosis on the loss of neurological abilities including movement, speech, and memory are now extensively recognized and better understood (Jokinen et al. 2015). Thus, methods aimed at antiapoptotic mechanisms could be beneficial for the treatment of cerebral ischemic stroke.

Mitochondria, a type of special cellular organelle, play crucial roles in energy production and calcium-homeostasis mediation. During ischemic stroke, the occlusion of blood vessels and insufficient oxygen supply to neurons induces fragmented mitochondria, thereby disturbing the ATP supply and resulting in excessive ROS accumulation (Wei et al. 2019). Excessive ROS production is related to the opening of mitochondrial permeability transition pore (mPTP) and the release of Cytochrome C and apoptosis-inducing factor (AIF) from mitochondria (Javadov and Kuznetsov 2013). The release of Cytochrome C and AIF activates caspases proteases through the apoptosome formation, eventually aggravating the tissue injury (Shakeri et al. 2017). Therefore, targeting mitochondrial health could be a promising therapeutic alternative candidate for ischemic injury.

Calenduloside E (CE) is a natural triterpenoid saponin and isolated from *Aralia elata* (Miq.) Seem (Araliaceae), (AS). AS are usually used as an antihypertensive, anti-arrhythmic and anti-diabetic agent in traditional Chinese medicine (Wang et al. 2014). In human umbilical vein endothelial cells (HUVECs), CE and CE analog can repress ox-LDL induced apoptosis (Tian et al. 2019). In H9c2 cardiomyocytes, CE and its analogs could alleviate H_2_O_2_-induced apoptosis (Tian et al. 2017). Additionally, CE can ameliorate myocardial ischemia/reperfusion (I/R) injury by regulating AMPK activation-mediated OPA1- related mitochondrial fusion (Wang et al. 2020a). However, the biological function of CE and its molecular mechanism has not been reported in ischemic/hypoxic neurological injury. In the present study, we investigated the effects and molecular mechanism of CE in ischemic/hypoxic neurological injury to provide an experimental basis for the treatment of ischemic stroke.

## Materials and methods

### Cell culture and treatment

The HT22 cells were purchased from Procell Life Science & Technology Co., Ltd. (Wuhan, China) and cultured in Dulbecco’s Modified Eagle’s Medium (DMEM; Gibco, Carlsbad, CA, USA) with 10% fetal bovine serum (FBS; Gibco, Carlsbad, CA, USA) at 37 °C. For oxygen-glucose deprivation (OGD) insult, the neurons were maintained in a hypoxic chamber (Thermo Fisher Scientific Inc., Waltham, MA, USA) at 94% N_2_, 1% O_2_, and 5% CO_2_ and then cultured in glucose-free DMEM solution at 37 °C for 0, 2, 4, 6, or 8 h, as previously described (Lai et al. 2020). After OGD, the neurons were cultured in normal DMEM solution with 10% FBS for 24 h. In the CE (MedChemExpress, NJ, USA) treatment groups, HT22 cells were pretreated with CE at doses of 1, 2, 4, or 8 µg/mL for 4 h prior to OGD/R (Tian et al. 2019; Wang et al. 2020a).

## Middle cerebral artery occlusion and reperfusion (MCAO/R) model and treatment

Animal experiments were approved by the ethics committee of Lanzhou University Second Hospital and conducted in accordance with the guidelines of the Institutional Animal Care and Use Committee of the Institute of Nutrition and Health. Mice were randomly divided into six groups: sham, MCAO, MCAO + CE 5 mg/kg (CE 5 mg/kg), MCAO + CE 10 mg/kg (CE 10 mg/kg), MCAO + CE 20 mg/kg (CE 20 mg/kg), MCAO + CE 30 mg/kg (CE 30 mg/kg). Focal brain ischemia was induced by MCAO/R in male C57BL/6J mice as described previously (Kuo et al. 2016; Yu et al. 2010). In a typical procedure, mice were anesthetized with ketamine (12 mg/kg) and xylazine (10 mg/kg) and the right carotid bifurcation was exposed to insert a silicone-coated 8 − 0 filament. The filament was inserted to the end of the internal carotid artery to block the origin of the right middle cerebral artery, thereby inducing the occlusion of the middle cerebral artery. A laser Doppler flowmetry (PerifluxSystem 5000; Perimed, Stockholm, Sweden) was used to detect the regional cerebral blood flow during surgery to confirm the successful occlusion. After 1 h of occlusion, the filament was withdrawn to recover the cerebral blood flow for 48 h. In the sham group, the right carotid arteries of mice were surgically exposed without subsequent MCAO. For CE treatment, 5, 10, 20 or 30 mg/kg CE in ultrapure water containing 0.5% sodium carboxymethylcellulose were administered by oral gavage as described previously (Wang et al. 2020a, b). Treatment groups were administered with CE at 1 h post-occlusion by oral gavage, and followed by oral gavage at 6, 12, 18, 24, 30, 36, and 42 h as described previously (Andrabi et al. 2019). The mice in the sham group were administered with an equal volume of ultrapure water containing 0.5% sodium carboxymethylcellulose.

## Neurobehavioral evaluation

Neurobehavioral evaluation was performed 48 h after reperfusion in accordance with a previously described method (Zhang et al. 2018). In a typical procedure, neurobehavioral evaluation was scored using a five-point scale: 0, no neurological deficit; 1, failure to extend left forepaw fully; 2, circling to the left; 3, inability to bear weight on the left; 4, no spontaneous walking with depressed level of consciousness.

## Infarct volume assessment

Infarct volume assessment was performed 48 h after reperfusion and determined by 2,3,5-triphenyltetrazolium chloride (TTC) staining. In a typical procedure, mice were euthanized under anesthesia, and their brains were rapidly removed and cut into 2 mm-thick slices. The slices were incubated with 2% TTC solution (Solarbio, Beijing, China) at 37 °C for 30 min. The image was photographed with a digital camera and the infarction volume was measured using Image J software (National Institutes of Health, Bethesda, MD, USA).

## Cell viability assay

Cell viability was determined by Cell Counting Kit-8 (CCK-8) assay. HT22 cells were cultured on 96-well plates at a density of 1 × 10^4^ cells/well for 24 h. After OGD for 0, 2, 4, 6, or 8 h and reperfusion for 24 h, the medium was removed and the cells were incubated with 10 µL of Cell Counting Kit-8 (CCK-8) solution (Beyotime, Shanghai, China) for 1 h at 37 °C. The absorbance of each well was measured at 450 nm using an automatic microplate reader (Bio-Tek M200, Tecan, Austria).

## Terminal deoxynucleotidyl transferase (TdT)-mediated dUTP nick-end labeling (TUNEL) assay

HT22 cells were seeded onto 24-well plates on glass coverslips at a density of 1 × 10^5^ cells/well for 24 h. After treatment as described above, the cells were fixed in 4% paraformaldehyde for 30 min at room temperature, washed for three times, and incubated in 0.3% Triton X-100 for 5 min. After washing, 50 µL of TUNEL solution (Roche Diagnostics, Indianapolis, IN, USA) was added to each well. After incubation for 1 h at 37 °C, HT22 cells were washed with PBS for three times, and DAPI was used to stain the cell nuclei. TUNEL-positive cells were counted from three fields under a fluorescence microscope at 200× magnification. The percentages of apoptotic cells were expressed as the ratio of TUNEL -positive cells to the DAPI-positive cells across the three fields (Kim and Lee 2014).

After treatment, mice were anesthetized and perfused through the heart with cold saline followed by 4% paraformaldehyde in phosphate buffer. Brains were removed and fixed in 4% paraformaldehyde at 4 °C overnight. Brain tissues embedded in paraffin were cut into 5 μm-thick sections. The sections were then deparaffinized and rehydrated. TUNEL solution (Roche Diagnostics, Indianapolis, IN, USA) was added to each section. After incubation for 1 h at 37 °C, the sections were washed with PBS for three times, and the neurons were stained with mouse anti-NeuN antibody (Proteintech Group, Inc., Wuhan, China). The sections were photographed using a microscope with a digital camera (Olympus, Tokyo, Japan). The collocation of green (TUNEL) and red (NeuN) indicated the apoptotic neurons. The percentages of TUNEL-positive neurons relative to the NeuN-positive cells were regarded as the percentages of apoptotic neurons. Five different fields (200× magnification) per section were selected, and then the number of TUNEL positive neurons and the number of neurons were then counted (Liu et al. 2020). The percentage of TUNEL positive neurons per mouse was obtained from three sections. Five mice per group were analyzed.

## Mitochondrial fission, mitochondrial ROS production and mitochondrial membrane potential (Δψm) assay

For mitochondrial fission assay, HT22 cells after treatment were washed with PBS and incubated with 200 nM MitoTracker® Deep Red FM (Yeasen, Shanghai, China) for 30 min at 37 °C. Mitochondrial morphology was visualized under a confocal microscope (LSM 750, Zeiss, Gottingen, Germany).

For mitochondrial ROS assay, HT22 cells after treatment were washed with PBS and incubated with 100 nM MitoTracker® Green FM (Yeasen, Shanghai, China) and 5 µM MitoSOX Red Mitochondrial Superoxide Indicator (Yeasen, Shanghai, China) for 10 min at 37 °C (Wu et al. 2017). HT22 cells were photographed using a confocal microscope.

Mitochondrial membrane potential (Δψm) was analyzed using tetramethylrhodamine ethyl ester perchlorate (TMRE) staining. HT22 cells after treatment were washed with PBS and incubated with TMRE (Beyotime, Shanghai, China) for 30 min at 37 °C. HT22 cells were photographed using a confocal microscope.

## Intracellular calcium assay

HT22 cells after treatment were washed three times with PBS and incubated with 2.5 µM Fluo-3AM (Beyotime, Shanghai, China) for 30 min at 37 °C. After washing, the cells were imaged by a microscope with a digital camera and the fluorescent intensity was analyzed using ImageJ software (National Institutes of Health, Bethesda, MD, USA) (Liao et al. 2018).

## Western blot

After treatment, HT22 cells and brain tissues were collected and lysed using ice-cold RIPA lysis buffer (Beyotime, Shanghai, China). The lysate was centrifuged at 12,000 g for 10 min at 4 °C. The protein concentrations were measured using a BCA protein assay kit (Beyotime, Shanghai, China). The supernatant was mixed with loading buffer (Beyotime, Shanghai, China) and denatured at 95 °C for 5 min. Proteins were separated by 10% SDS-PAGE and transferred onto PVDF membranes. The PVDF membranes were blocked in TBST containing 5% BSA and incubated with the following primary antibodies: rabbit anti-Drp1 (1:2,000, 12957-1-AP, Proteintech, Wuhan, China), rabbit anti-p-Drp1 (ser 637; ab193216, 1:1,000, Abcam, Cambridge, MA, USA), rabbit anti-COX4 (1:5,000, 11242-1-AP, Proteintech, Wuhan, China), rabbit anti-GAPDH (1:2,000, 10494-1-AP, Proteintech, Wuhan, China), rabbit anti-Cytochrome C (1:4,000, 10993-1-AP, Proteintech, Wuhan, China), rabbit anti-Bax (1:5,000, 50599-2-Ig, Proteintech, Wuhan, China), rabbit anti-Bcl-2 (1:2,000, 26593-1-AP, Proteintech, Wuhan, China), rabbit anti-Caspase3 (1:1,000, 19677-1-AP, Proteintech, Wuhan, China) and rabbit anti-Caspase9 (1:500, 10380-1-AP, Proteintech, Wuhan, China). After washing three times with TBST, the membranes were incubated with goat anti-rabbit secondary antibody (1:5,000, SA00001-2, Proteintech, Wuhan, China). After washing, the corresponding antigen-antibody complexes were revealed using enhanced chemiluminescence reagents (Pierce Biotech, IL, USA).

To prepare mitochondrial protein samples, mitochondria from the peri-ischemic cortex and hippocampus of mice were isolated with a Tissue Mitochondria Isolation Kit (Beyotime, Shanghai, China) according to the manufacturer’s instruction. In a typical procedure, brain tissues were collected, divided into small pieces, and homogenized in the mitochondrial separation reagent A on an ice bath. After homogenization, the samples were centrifuged at 600 g for 5 min at 4 °C. The supernatant was collected and further centrifuged at 11,000 g for 10 min at 4 °C. The precipitate was isolated mitochondria. Mitochondria from HT22 cells were isolated with a Cell Mitochondria Isolation Kit (Beyotime, Shanghai, China) according to the manufacturer’s instruction. In a typical procedure, HT22 cells after treatment were washed with PBS, collected, and centrifuged at 600 g for 5 min at 4 °C. HT22 cells were incubated with the mitochondria isolation reagent containing 1 mM PMSF for 15 min. After homogenizing the mixture for 30 times and centrifuging at 600 g for 10 min at 4 °C, the supernatant was collected and further centrifuged at 11,000 g for 10 min at 4 °C. The precipitate was isolated mitochondria. Mitochondria were lysed with a cold mitochondrial protein extraction kit (KeyGEN Biotech, Nanjing, China) to obtain mitochondrial proteins. The mitochondrial and cytosolic fractions proteins were measured as described above.

### Statistical analysis

All data were analyzed using SPSS 19.0 software (IBM Corp., Chicago, IL, USA) and expressed as the mean ± SD. Significant differences between groups were analyzed using Student’s *t* test or one-way ANOVA. *P* < 0.05 was regarded statistically significant.

## Results

### CE alleviated OGD/R-induced neuronal injury in HT22 cells

**Fig. 1:**
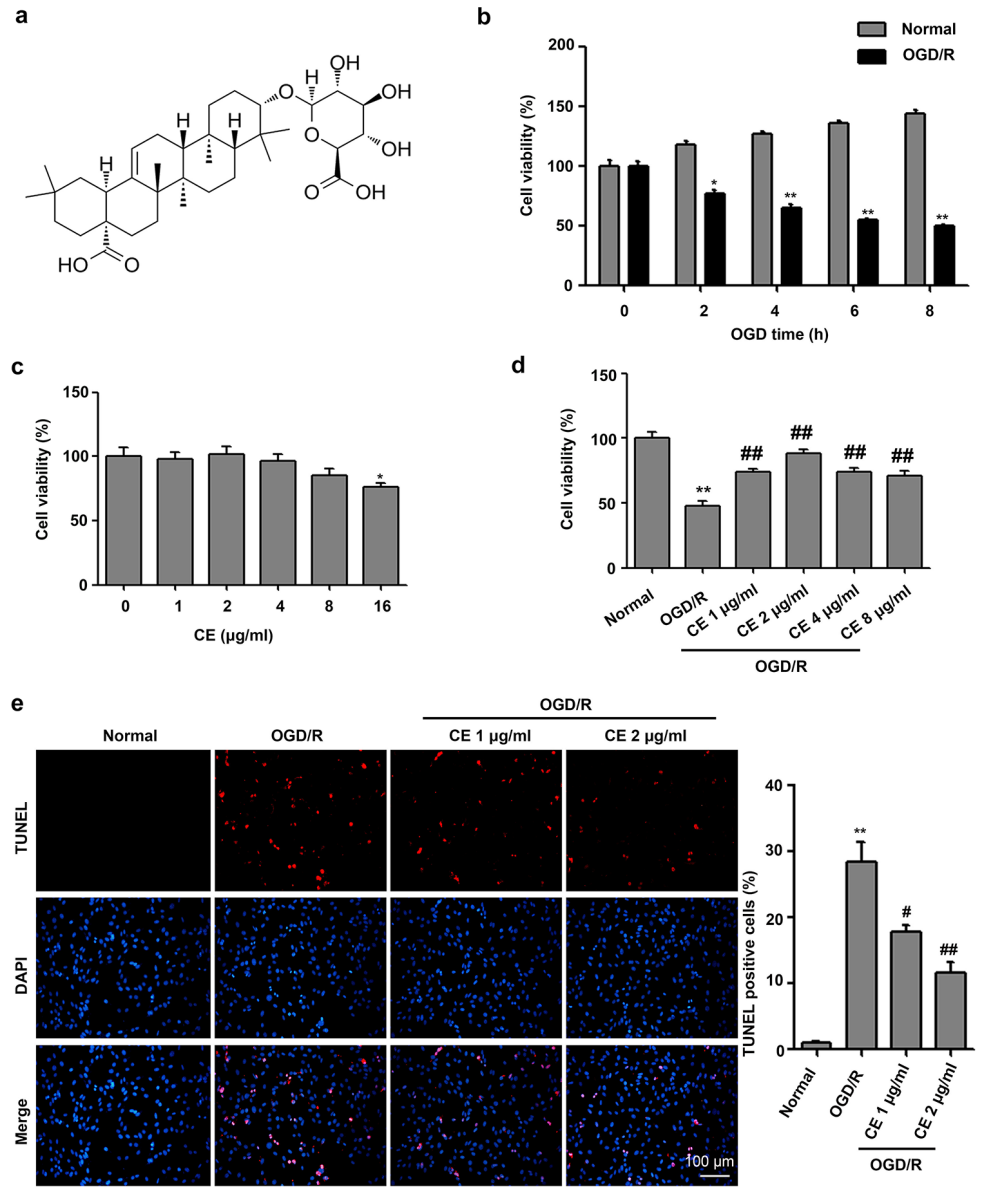
CE ameliorated OGD/R-induced neuronal injury in HT22 cells. **a** CE structure. **b** For OGD, HT22 cells were cultured in glucose-free DMEM solution and maintained in a hypoxic chamber at 94% N_2_, 1% O_2_, and 5% CO_2_ for 0, 2, 4, 6, or 8 h at 37 °C. After OGD, the neurons were cultured in complete medium for 24 h. Cell viability was measured using CCK-8 (n = 3). **c** HT22 cells were treated with CE at doses of 0, 1, 2, 4, 8, or 16 µg/mL for 4 h. Cell viability was measured using CCK-8 (n = 3). **d** HT22 cells were pretreated with CE at doses of 1, 2, 4, or 8 µg/mL for 4 h prior to OGD/R. After OGD for 4 h and reperfusion for 24 h, cell viability was measured using CCK-8 (n = 3). **e** HT22 cells were pretreated with CE at doses of 1 and 2 µg/mL for 4 h prior to OGD/R. After OGD for 4 h and reperfusion for 24 h, the cell apoptosis of HT22 cells was determined by TUNEL (n = 3, scale bar = 100 μm). *P < 0.05 and **P < 0.01 compared with the normal group. ^#^*P* < 0.05 and ^##^*P* < 0.01 compared with the OGD/R group

The structure of CE is shown in Fig. 1a. To determine the effect of CE on HT22 cells, we established an OGD/R model in vitro. After exposing HT22 cells to OGD/R, cell viability was decreased with increased OGD treatment time (Fig. 1b). The cell viability of HT22 cells after 4 h was 51.2%, whereas the cell viability of HT22 cells after 6 h OGD was 40.7%. Therefore, 4 h was selected as the optimum OGD treatment time. To determine the toxic effect of CE on HT22 cells, different concentrations of CE (0, 1, 2, 4, 8, or 16 µg/mL) were used to treat HT22 cells. As shown in Fig. 1c, when the concentration of CE was lower than 16 µg/mL, CE had no toxic effect on cells. To determine the protective effect of CE on HT22 cells, HT22 cells were pretreated with CE at doses of 0, 1, 2, 4, and 8 µg/mL for 4 h prior to OGD/R. As shown in Fig. 1d, cell viability in the OGD/R group was significantly lower than that in the normal group, and cell viability was significantly reversed by the pretreatment of CE with increased dosage. CE at doses of 1 µg/mL and 2 µg/mL exhibited the most significant protective effect, so 1 µg/mL and 2 µg/mL were selected for further experiments. Subsequently, we determined if CE could improve OGD/R-induced apoptosis. Our results showed that the proportion of apoptosis in HT22 cells after OGD/R significantly increased compared with that in the normal group, and CE at doses of 1 µg/mL and 2 µg/mL significantly decreased OGD/R-induced neuronal apoptosis (Fig. 1e).

## CE alleviated OGD/R-induced excessive mitochondrial fission in the HT22 cells

**Fig. 2:**
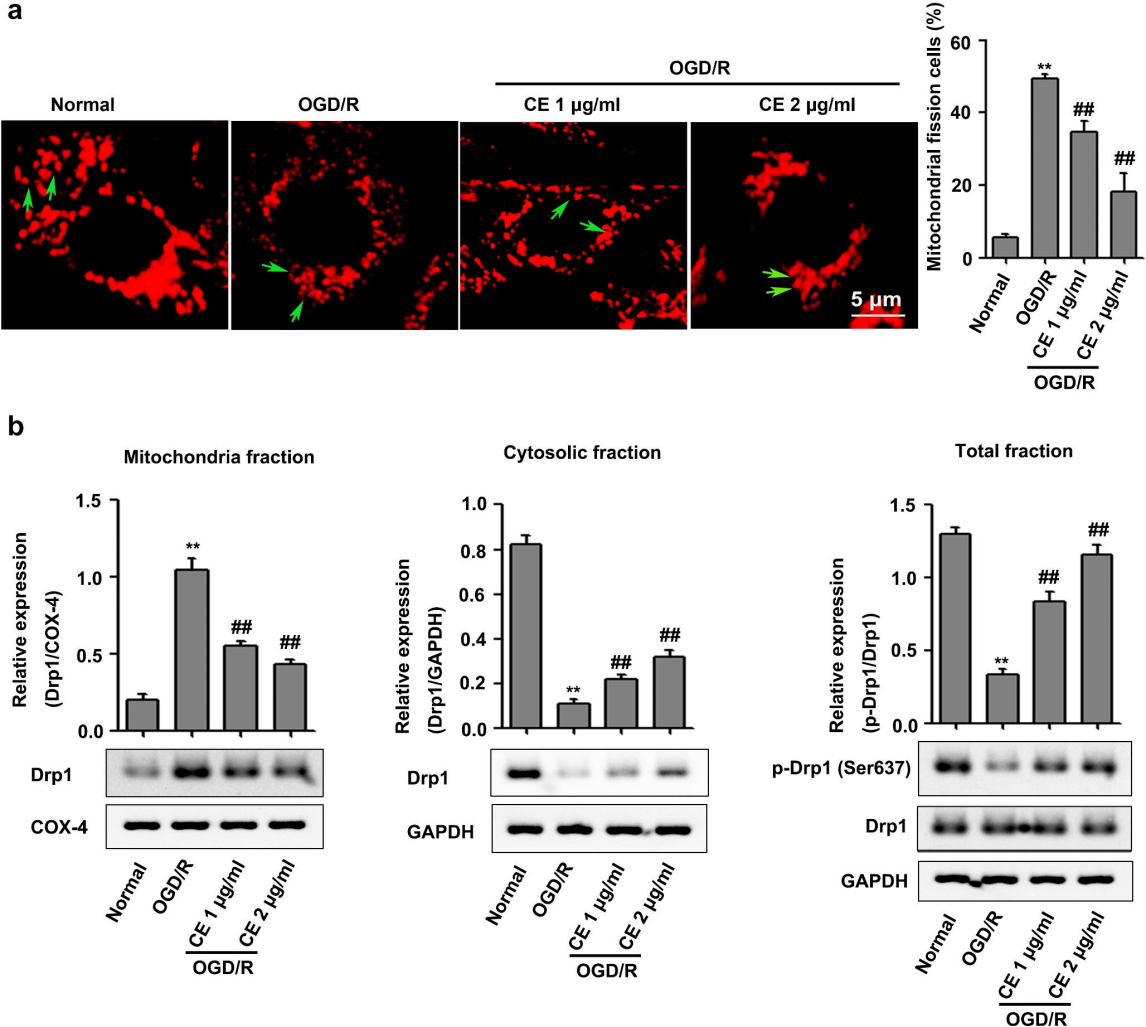
CE inhibited OGD/R-induced excessive mitochondrial fission in HT22 cells. **a** HT22 cells were pretreated with CE at doses of 1 and 2 µg/mL for 4 h prior to OGD/R. After OGD for 4 h and reperfusion for 24 h, mitochondrial morphology was determined with MitoTracker® Deep Red FM (n = 3, scale bar = 5 μm). **b** The expression of Drp1 in the mitochondrial and cytosolic fractions and total levels of Drp1 and p-Drp1 (Ser637) were determined by Western blot (n = 3). **P < 0.01 compared with the normal group. ^##^*P* < 0.01 compared with the OGD/R group

CE can reportedly alleviate mitochondrial injury by regulating mitochondrial fission and fusion proteins (Wang et al. 2020a). An imbalance between mitochondrial fusion and fission results in abnormal mitochondrial structure, which is related to neuronal apoptosis during cerebral I/R injury (Zhang et al. 2013). Thus, we determined the effect of CE on mitochondrial structure in HT22 cells after OGD/R treatment. Results showed that mitochondria in the HT22 cells had a flexible reticular formation in the normal group, whereas OGD/R treatment caused mitochondrial fragmentation in HT22 cells, a morphological change referred to as mitochondrial fission. The proportion of mitochondrial-fission neurons after OGD/R was significantly increased compared with that in the normal group, whereas CE treatment significantly alleviated OGD/R-induced mitochondrial fission in HT22 cells (Fig. 2a). Moreover, CE inhibited mitochondrial Drp1 recruitment (Wang et al. 2020a). Thus, we determined whether CE alleviated OGD/R-induced mitochondrial fission by inhibiting mitochondrial Drp1 recruitment. As shown in Fig. 2b, OGD/R induced the translocation of Drp1 from cytoplasm to mitochondria, which was reduced by CE treatment. Increased Drp1 Ser637 phosphorylation has been shown to contribute to decreased mitochondrial fission (Li et al. 2017). Consistently, the results showed that OGD/R treatment significantly inhibited Drp1 Ser637 phosphorylation in HT22 cells, whereas CE treatment significantly improved the inhibition of Drp1 Ser637 phosphorylation induced by OGD/R.

## CE alleviated OGD/R-induced mitochondrial dysfunction in HT22 cells

**Fig. 3:**
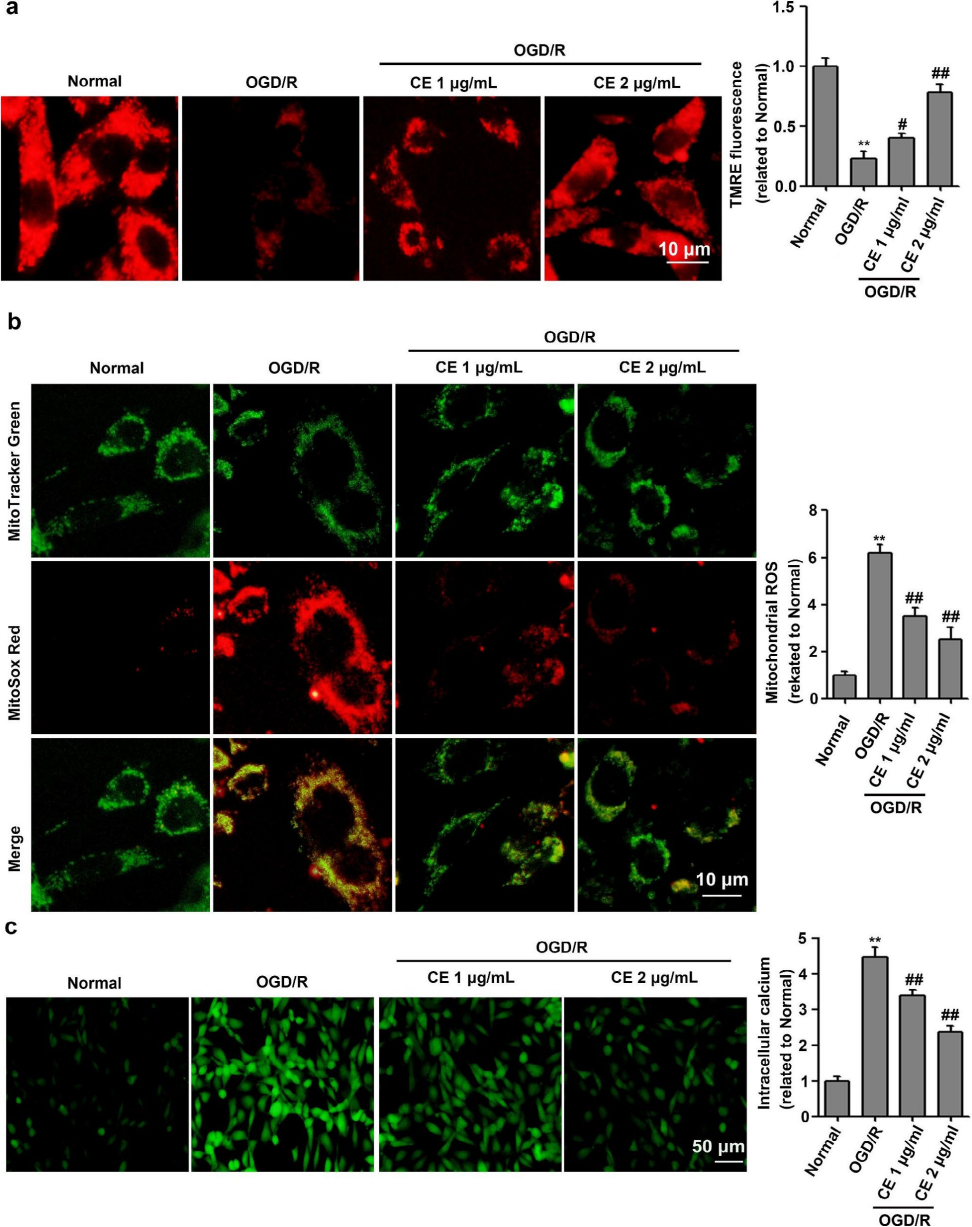
CE ameliorated OGD/R-induced mitochondrial dysfunction in HT22 cells. **a** HT22 cells were pretreated with CE at doses of 1 and 2 µg/mL for 4 h prior to OGD/R. After OGD for 4 h and reperfusion for 24 h, mitochondrial membrane potential (Δψm) was determined with by TMRE staining (n = 3). Scale bar, 10 μm. **b** For mitochondrial ROS assay, HT22 cells were stained for mitochondria (green) and ROS (red) (n = 3). Scale bar, 10 μm. **c** For intracellular calcium assay, HT22 cells were stained with Fluo-3AM (n = 3). Scale bar, 50 μm. **P < 0.01 compared with the normal group. ^##^*P* < 0.01 compared with the OGD/R group

Since CE treatment could alleviate OGD/R-induced mitochondrial integrity, we further investigated the effect of CE on mitochondrial dysfunction, which was characterized by mitochondrial membrane potential (Δψm) collapse, mitochondrial calcium overload, and excessive ROS production. As shown in Fig. 3a, OGD/R treatment significantly induced mitochondrial membrane potential (Δψm) collapse in HT22 cells, whereas CE treatment prevented the drop of TMRE signal. Furthermore, we found that OGD/R treatment significantly induced mitochondrial ROS generation and cellular calcium accumulation in HT22 cells, whereas CE treatment significantly alleviated these alterations (Fig. 3b and c).

## CE treatment alleviated mitochondrial-associated apoptosis in HT22 cells after OGD/R

**Fig. 4:**
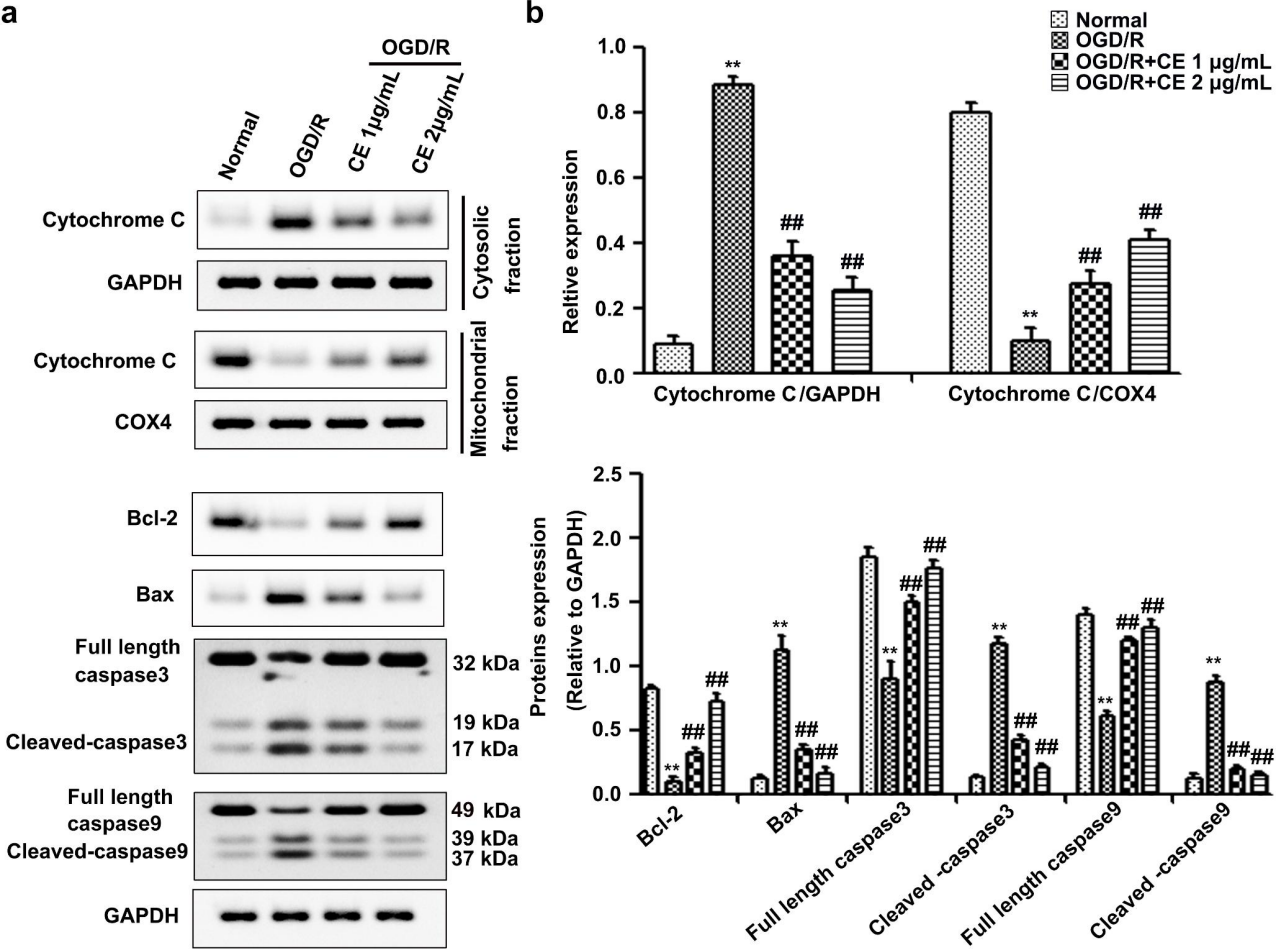
CE treatment repressed mitochondrial-associated apoptosis in HT22 cells after OGD/R. **a** HT22 cells were pretreated with CE at doses of 1 and 2 µg/mL for 4 h prior to OGD/R. After OGD for 4 h and reperfusion for 24 h, HT22 cells were collected, and the expression of Cytochrome C in the mitochondrial and cytosolic fractions was determined using Western blot. The total levels of Bcl-2, Bax, full length Caspase3, Cleaved-caspase3, full length Caspase9 and Cleaved-caspase9 were also determined using Western blot. **b** Histogram showing quantification of images in (**a)** (n = 3). **P < 0.01 compared with the normal group. ^##^*P* < 0.01 compared with the OGD/R group

ROS production and Ca^2+^ overload as mitochondrial events could induce Cytochrome C release from mitochondria to cytosol, thereby activating the apoptotic cascade (Andrabi et al. 2019). To determine whether CE treatment alleviated OGD/R-induced neuronal apoptosis by regulating mitochondrion-dependent apoptosis, we analyzed the associated apoptotic markers Cytochrome C, Bcl-2, Bax, Cleaved-caspase3, Cleaved-caspase9 and full length of caspase3 and caspase9. As shown in Fig. 4a and b, OGD/R treatment significantly induced Cytochrome C release from mitochondria to cytosol, whereas CE treatment significantly reversed these alterations. Additionally, Bcl-2 and full length of caspase3 and caspase9 expression decreased, and Bax, Cleaved-caspase3 and Cleaved-caspase9 expression significantly increased in HT22 cells after OGD/R treatment, which could be remarkably reversed by pretreatment with CE (Fig. 4a and b).

## CE treatment ameliorated neurological deficits and brain injury in mice after MCAO

**Fig. 5:**
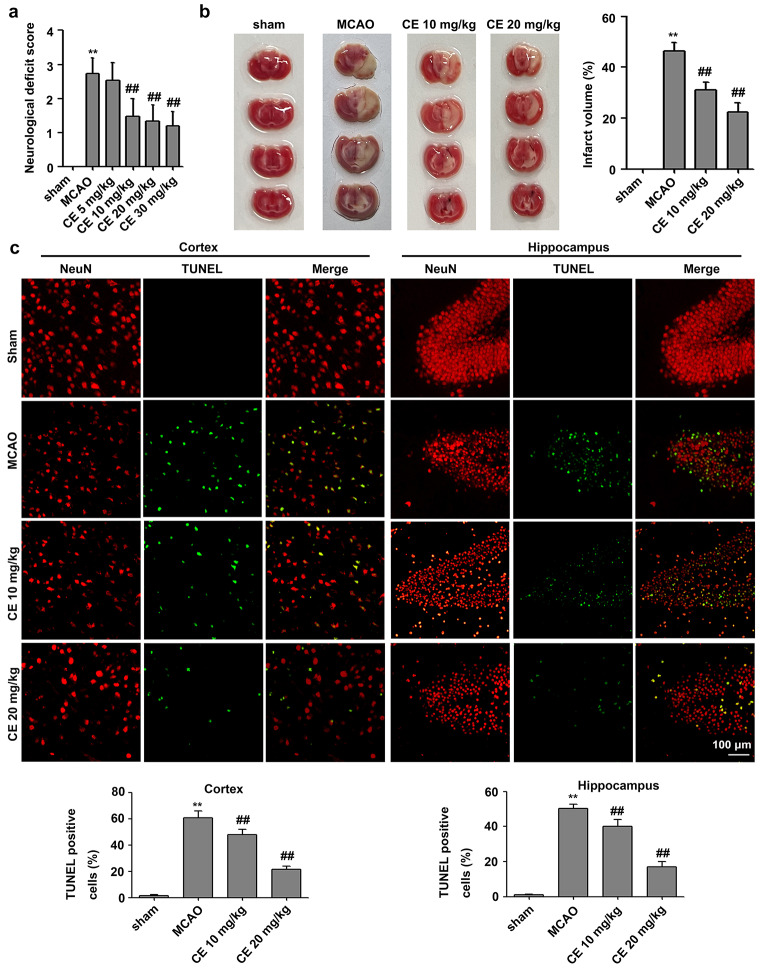
CE treatment ameliorated stroke outcomes. **a** Mice were subjected to MCAO for 1 h and followed by 48 h of reperfusion. For CE treatment, 5, 10, 20 or 30 mg/kg CE in ultrapure water containing 0.5% sodium carboxymethylcellulose were administered by oral gavage 1 h post-occlusion and followed oral gavage at 6, 12, 18, 24, 30, 36, and 42 h post-occlusion. Neurological function was measured using the neurological deficit score (n = 15). **b** TTC analysis of brain sections was performed to determine the infarct volume in mice after MCAO surgery (n = 5). **c** TUNEL staining was performed to determine MCAO-induced neuronal apoptosis in the peri-infarct region of the cortex and hippocampus of mice. Sections were stained for NeuN (red) and TUNEL (green) (n = 5). Scale bar, 100 μm. **P < 0.01 compared with the sham group. ^##^*P* < 0.01 compared with the MCAO group

To further confirm the neuroprotective action of CE during ischemic stroke, we established an MCAO model in mice and evaluated the effect of CE on cerebral injury in mice 48 h after MCAO surgery. As shown in Fig. 5a, MCAO markedly induced neurological deficits, and CE treatment at doses of 10, 20, and 30 mg/kg significantly ameliorated neurological function. However, CE treatment at doses of 5 mg/kg did not affect the MCAO-induced neurological deficits. Our results showed that CE treatment at doses of 30 mg/kg did not ameliorate neurological function when compared with CE treatment at doses of 10 mg/kg or 20 mg/kg. Thus, CE treatment at doses of 10 and 20 mg/kg was selected for further experiments. Moreover, a TTC analysis of brain sections was performed to determine the infarct volume in mice after MCAO surgery. Results showed that infarct volume in mice after MCAO surgery was significantly increased, whereas the infarct volume in CE-treated groups was significantly lower than that in the MCAO group (Fig. 5b). Moreover, TUNEL staining was performed to determine the effect of CE treatment on MCAO-induced neuronal apoptosis. Results showed that neuronal apoptosis in the peri-infarct region of cortex and hippocampus at 48 h after MCAO was significantly increased compared with that in the sham group, and CE treatment at doses of 10 and 20 mg/kg significantly ameliorated MCAO-induced neuronal apoptosis (Fig. 5c).

## CE treatment protected mitochondria in mice after MCAO

**Fig. 6:**
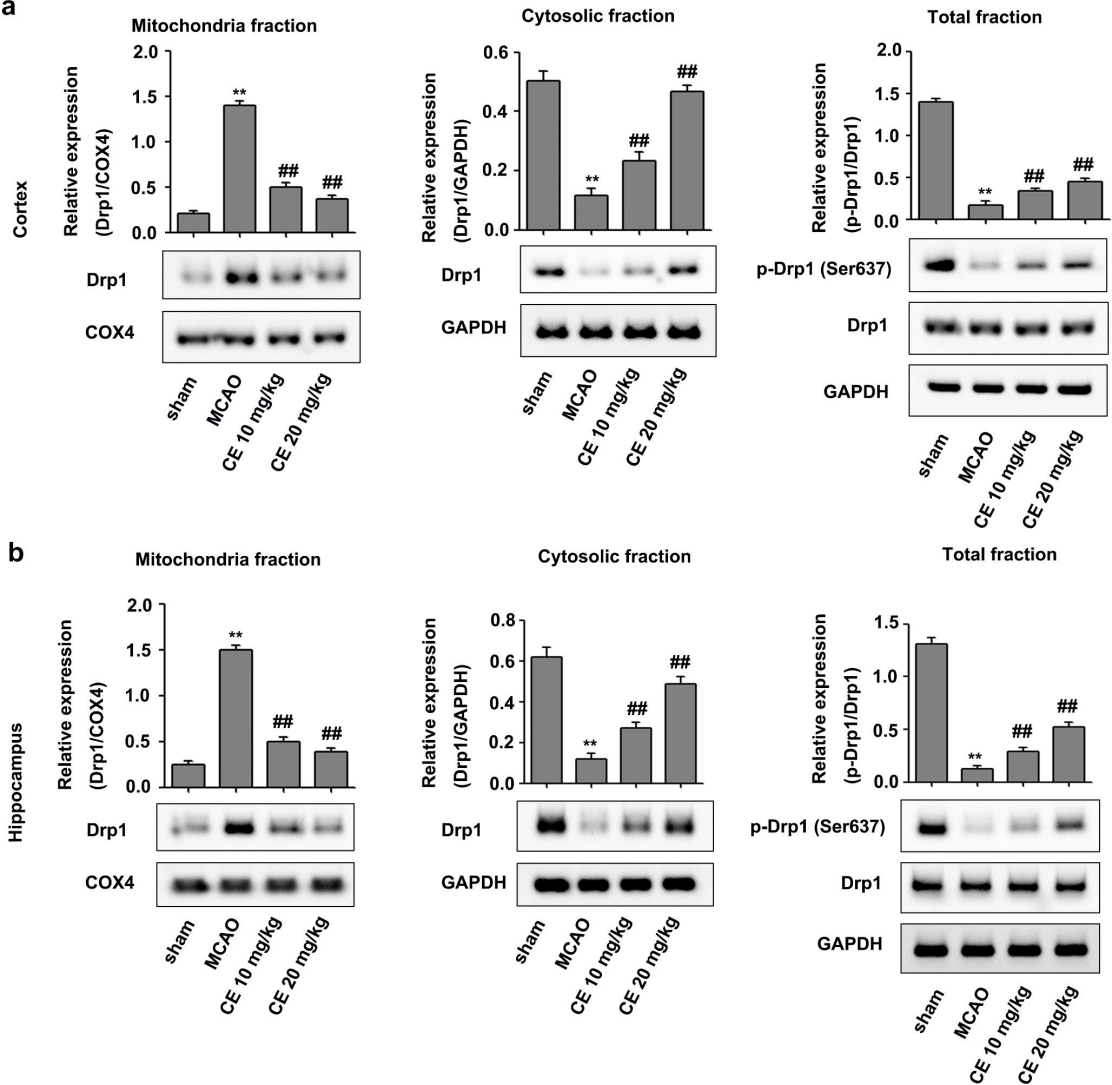
CE treatment ameliorated MCAO-induced excessive mitochondrial fission in the peri-infarct region of the cortex and hippocampus of mice during stroke. The mice were subjected to MCAO for 1 h and followed by 48 h of reperfusion. For CE treatment, 10 and 20 mg/kg CE in ultrapure water containing 0.5% sodium carboxymethylcellulose were administered by oral gavage 1 h post-occlusion and followed oral gavage at 6, 12, 18, 24, 30, 36, and 42 h post-occlusion. **a** and **b** Western blot was used to determine the effects of CE on Drp1 expression in the mitochondrial and cytosolic fractions and the total levels of Drp1 and p-Drp1 (Ser637) in brain tissues from the peri-infarct region of the cortex (**a**) and hippocampus (**b**) after MCAO (n = 4). **P < 0.01 compared with the sham group. ^##^*P* < 0.01 compared with the MCAO group

To validate the protection of CE on OGD/R-induced mitochondrial injury in HT22 cells, Western blot was performed in the peri-infarct region of cortex and hippocampus tissues from mice after MCAO surgery. As shown in Fig. 6a and b, MCAO induced the translocation of Drp1 from cytoplasm to mitochondria, which was reduced by CE treatment. Furthermore, MCAO treatment significantly inhibited Drp1 Ser637 phosphorylation in the peri-infarct region of cortex and hippocampus tissues, whereas CE treatment significantly improved the inhibition of Drp1 Ser637 phosphorylation induced by MCAO.

## CE treatment alleviated mitochondrial-associated apoptosis in mice after MCAO

**Fig. 7:**
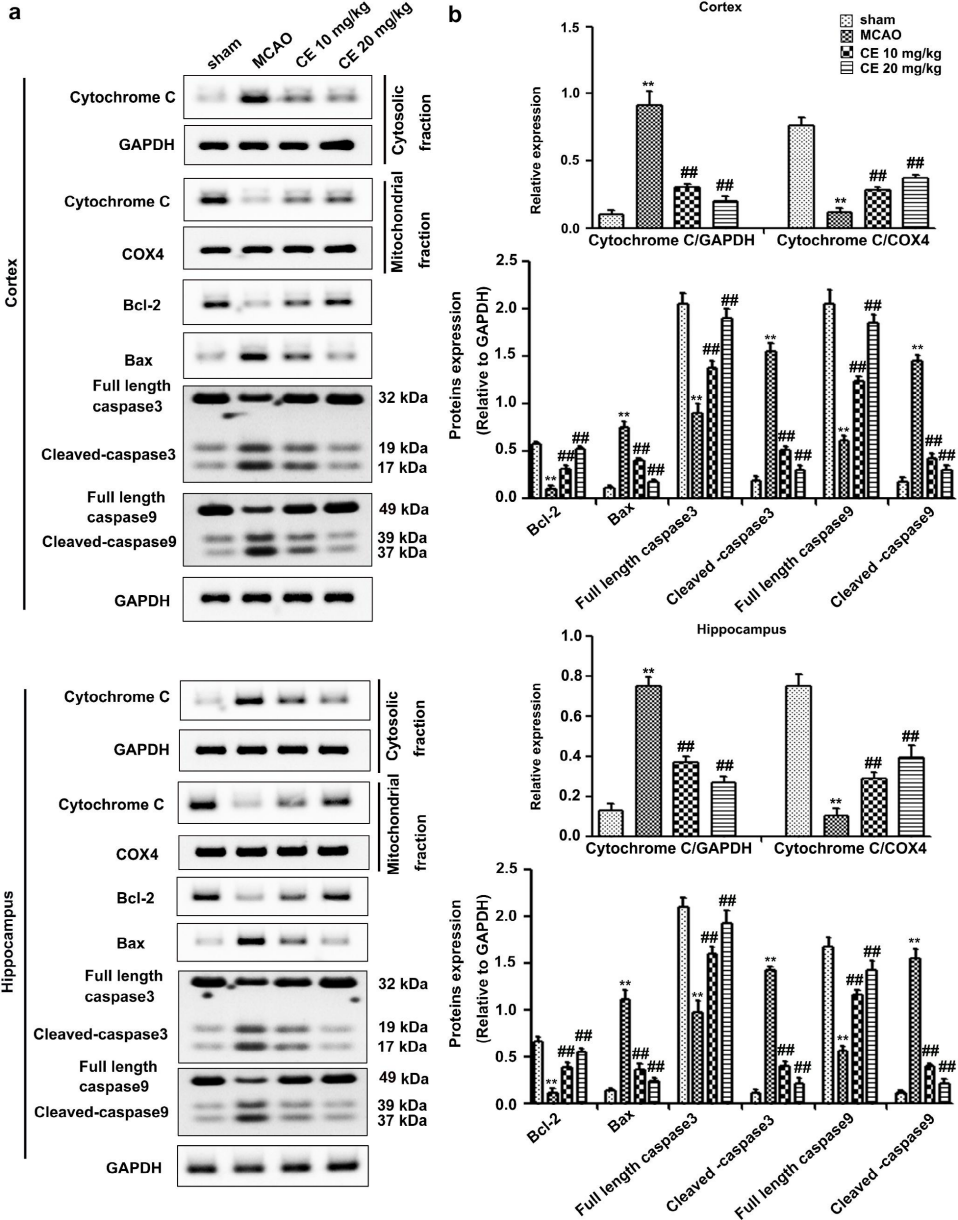
CE treatment inhibited mitochondrial-associated apoptosis in mice after MCAO. **a** Western blot was used to determine the expression of Cytochrome C in the mitochondrial and cytosolic fractions and the total levels of Bcl-2, Bax, full length caspase3, Cleaved-caspase3, full length caspase9 and Cleaved-caspase9 in the peri-infarct region of the cortex and hippocampus from mice subjected to the indicated treatment. **b** Histogram showing quantification of images in (**a)** (n = 4). **P < 0.01 compared with the sham group. ^##^*P* < 0.01 compared with the MCAO group

To validate the effects of CE on OGD/R-induced mitochondria-dependent apoptosis in HT22 cells, Western blot was performed to detect the associated apoptotic markers Cytochrome C, Bcl-2, Bax, Cleaved-caspase3, Cleaved-caspase9, and full length of caspase3 and caspase9 in the peri-infarct region of cortex and hippocampus tissues from mice after MCAO surgery. As shown in Fig. 7a and b, MCAO significantly induced Cytochrome C release from mitochondria to cytosol, whereas CE treatment significantly reversed these alterations. Additionally, Bcl-2 and full length of caspase3 and caspase9 expression decreased and Bax, Cleaved-caspase3 and Cleaved-caspase9 expression significantly increased in the peri-infarct region of cortex and hippocampus tissues from mice after MCAO surgery, which was remarkably reversed by pretreatment with CE (Fig. 7a and b).

## Discussion

CE, one of the primary natural pentacyclic triterpenoid saponins in *Aralia elata* (Miq.) Seem (Araliaceae) (AS), is usually used as an antihypertensive, antiarrhythmic and antidiabetic agent in traditional Chinese medicine (Wang et al. 2014). The total saponins from AS have been successfully used for coronary heart disease treatment and completed phase III clinical trials in China (Wang et al. 2014). Previous studies have demonstrated that CE has an antiapoptosis effect under pathological conditions (Tian et al. 2017, 2019). For instance, CE and CE analogs can ameliorate ox-LDL-induced apoptosis in HUVECs (Tian et al. 2019). CE and its analogs can also alleviate H_2_O_2_-induced apoptosis in H9c2 cardiomyocytes (Tian et al. 2017). Thus, CE may ameliorate neuronal injury during an ischemic stroke. As expected, our results showed that CE treatment significantly ameliorated the OGD/R-induced the inhibition of cell viability and apoptosis in HT22 cells (Fig. 1d and e). To confirm the effect of CE on neurological injury in vivo, we performed an MCAO model in mice. We found that CE treatment significantly ameliorated neurological function and significantly reduced infarct volume (Fig. 4a and b). We also found that CE treatment significantly inhibited MCAO-induced neuronal apoptosis in the peri-infarct region of cortex and hippocampus tissues from mice after MCAO surgery (Fig. 4c). Consistent with our findings, Wang et al. found that CE can ameliorate myocardial I/R injury by reversing I/R-induced cardiac dysfunction and inhibiting infarct size (Wang et al. 2020a). Interestingly, our results also showed that CE treatment at doses of ≥ 16 µg/mL inhibited cell viability in HT22 cells (Fig. 1c). Consistent with our findings, Tang et al. found that CE below 20 µg/mL reduced LPS-induced inflammatory response in RAW264.7 cells, whereas CE at doses of ≥ 20 µg/mL inhibited the viability of RAW264.7 cells (Tang et al. 2019). Thus, appropriate concentration is very important for the protective effect of CE in ischemic stroke. Interestingly, a previous study has shown that CE treatment significantly inhibited HepG2 cell proliferation and migration by activating the p38/JNK-HMGB1 pathway (Wang et al. 2021). However, CE treatment did not affect the phosphorylation levels of p38 and JNK in RAW264.7 cells with or without LPS treatment (Tang et al. 2019). Thus, CE may have diverse functions under different pathological conditions.

In the present study, we showed that CE exerted protective effects on I/R injury during stroke, but its underlying mechanism remains unclear. Interestingly, a previous study has reported that CE can ameliorate myocardial I/R injury by inhibiting mitochondrial fission (Wang et al. 2020a). Mitochondria are highly dynamic cellular organelles characterized by fission, fusion, and transport to strategic locations (Liu et al. 2018a). This dynamic process is essential for the morphology, the subcellular location, and function of mitochondria, and excessive mitochondrial fission is associated with a number of neurological diseases (Qi et al. 2013). Furthermore, mitochondrial fission is an early event required for ischemic neuronal death, and the inhibition of mitochondrial fission is useful for ameliorating I/R injury during stroke (Martorell-Riera et al. 2014; Xu et al. 2017). Thus, CE may ameliorate I/R injury by regulating mitochondrial fission. As expected, we found that CE treatment significantly inhibited OGD/R-induced mitochondrial fission in HT22 cells (Fig. 2a). Previous studies have showed that mitochondrial fission protein Drp1 is involved in mediating mitochondrial fission (Qi et al. 2013; Xu et al. 2017). The inhibition of mitochondrial Drp1 recruitment reduces mitochondrial fission and maintained normal mitochondrial morphology and function in ischemic neurons (Zhou et al. 2017). Our results showed that CE significantly inhibited mitochondrial Drp1 recruitment in OGD/R-treated HT22 cells (Fig. 2b) and MCAO-treated mice (Fig. 6). Consistent with our results, CE can inhibit mitochondrial Drp1 recruitment in myocardial I/R injury (Wang et al. 2020a). Therefore, CE may inhibit mitochondrial fission by regulating mitochondrial Drp1 recruitment in I/R injury during stroke. Additionally, Drp1 phosphorylation at Ser637 inhibits Drp1 to the constriction sites of mitochondria, thereby preventing mitochondrial fission (Chang and Blackstone 2017). Our results showed that CE treatment significantly improved the inhibition of Drp1 Ser637 phosphorylation induced by OGD/R in vitro (Fig. 2b) and MCAO in vivo (Fig. 6). Thus, CE may inhibit mitochondrial Drp1 recruitment by increasing Drp1 Ser637 phosphorylation, thereby ameliorating mitochondrial fission in I/R injury during stroke.

Mitochondrial dysfunction characterized by ROS generation, calcium accumulation, opening of mitochondrial permeability transition pore (mPTP) and releasing of Cytochrome C plays an important role in apoptotic cell death during the I/R injury (Vosler et al. 2009). Following ischemia, persistent mPTP opening results in Ca^2+^ overload, thereby leading to mitochondrial depolarization and decreased in mitochondrial membrane potential (Rizzuto et al. 2012). Mitochondrial depolarization leads to excessive ROS production, and excessive accumulation of ROS and calcium in the mitochondria triggers a vicious cascade of mitochondrial damage, thereby leading to the apoptotic death of brain cells (He et al. 2020). Thus, ameliorating the mitochondrial dysfunction may be a promising therapeutic strategy for ischemic stroke. In addition, a recent report showed that CE alleviates calcium overload by promoting the interaction between LTCC and Bcl2-associated athanogene 3 (BAG3), thereby ameliorating myocardial I/R injury (Wang et al. 2020b). Our results showed that CE could alleviate mitochondrial membrane-potential collapse, mitochondrial ROS generation, and cellular calcium accumulation in HT22 cells after OGD/R treatment (Fig. 3). These results suggested that the protective effects of CE on I/R-induced neuronal injury were related to improved mitochondrial integrity. Moreover, persistent mPTP opening resulted in Cytochrome C release from mitochondria to cytosol, which in turn activated the apoptotic cascade involving caspases-9 and caspases-3 (He et al. 2020). Since CE treatment could improve mitochondrial integrity, CE may inhibit Cytochrome C release from mitochondria to cytosol. As expected, we found that CE treatment significantly inhibited Cytochrome C release from mitochondria to cytosol during ischemic stroke. We further determined the effects of CE on the proapoptotic protein Bax and antiapoptotic protein Bcl-2, both of which are related to the permeability of the mitochondrial membrane, the opening of the channels and release of Cytochrome C, as well as the intrinsic mitochondrial apoptotic program in the ischemia-induced death of astrocytes and neurons (Juurlink and Hertz 1993). We showed that CE treatment significantly reversed the increase in Bax, Cleaved-caspase3 and Cleaved-caspase9 protein levels and the decrease in Bcl-2, full length of caspase3 and caspase9 protein levels induced by cerebral I/R (Figs. 4 and 7). Overall, our results suggested that the protective effects of CE on I/R-induced neuronal injury were associated with the amelioration of mitochondrial apoptotic brain damage.

In summary, our results demonstrated that CE treatment can provide protective effects on cerebral I/R injury by ameliorating mitochondrial-associated apoptosis induced by excessive mitochondrial fission and mitochondrial dysfunction.
